# Accurate Binding Free Energy Method from End-State MD Simulations

**DOI:** 10.1021/acs.jcim.2c00601

**Published:** 2022-08-16

**Authors:** Ebru Akkus, Omer Tayfuroglu, Muslum Yildiz, Abdulkadir Kocak

**Affiliations:** † Department of Bioengineering, Gebze Technical University, 41400 Gebze, Kocaeli, Turkey; ‡ Department of Molecular Biology and Genetics, Gebze Technical University, 41400 Gebze, Kocaeli, Turkey; § Department of Chemistry, Gebze Technical University, 41400 Gebze, Kocaeli, Turkey

## Abstract

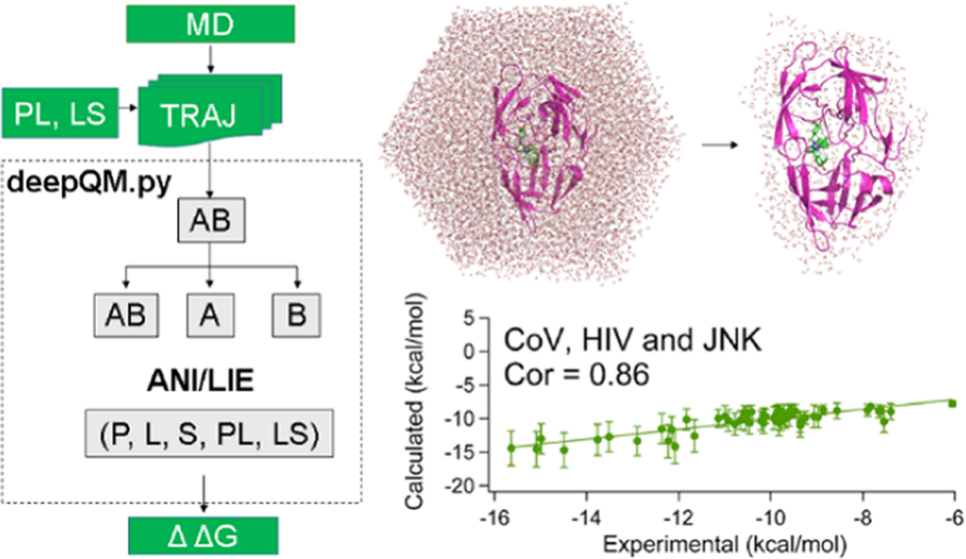

Herein, we introduce a new strategy to estimate binding free energies using end-state molecular dynamics simulation trajectories. The method is adopted from linear interaction energy (LIE) and ANI-2x neural network potentials (machine learning) for the atomic simulation environment (ASE). It predicts the single-point interaction energies between ligand–protein and ligand–solvent pairs at the accuracy of the wb97x/6-31G* level for the conformational space that is sampled by molecular dynamics (MD) simulations. Our results on 54 protein–ligand complexes show that the method can be accurate and have a correlation of *R* = 0.87–0.88 to the experimental binding free energies, outperforming current end-state methods with reduced computational cost. The method also allows us to compare BFEs of ligands with different scaffolds. The code is available free of charge (documentation and test files) at https://github.com/otayfuroglu/deepQM.

## Introduction

Developing small compounds that target medically relevant protein is one of the main strategies in modern drug discovery studies. There are two core questions that must be addressed in these efforts: (a) what is the binding mode? (i.e., where does it bind from the receptor?) and (b) what is the binding strength? (binding free energy).^1^ Inhibition of an enzyme can be involved in various mechanisms. For instance, an inhibitor can bind to the enzyme from the active site via competitive inhibition or it can bind from an exo/allosteric site by uncompetitive/noncompetitive inhibition.^2^

Kinetic studies conducted to understand the activity loss of the enzyme on a substrate by the inhibitor report values such as inhibition equilibrium constant *K*_i_ or half maximal inhibitory concentration IC_50_. These two experimental parameters are proportional to each other for competitive inhibition (they are equal in special cases).^2,3^ Thermodynamic equilibrium constant *K*_i_ and thus IC_50_ can be approximated to the experimental binding free energy of the system for the competitive inhibitors by
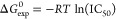
A more correct form is
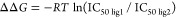
where ΔΔ*G* is the relative binding free energy between two competitive inhibitors. Δ*G* can also be calculated from the thermodynamic potential. In principle, it is possible to compare experimental binding free energies determined from IC_50_ and theoretically determined from IC_50_ predicted by thermodynamic potentials using computational methods for a series of competitive inhibitors. Thus, numerous efforts have been made to develop new strategies that can accurately predict binding free energies. However, a trade-off between computational accuracy and speed must be made.

Methods to calculate the potential binding free energy range from simple end-state methods such as linear response approximation (LRA) to linear interaction energy (LIE) and molecular mechanics Poisson–Boltzmann/generalized Born surface area (MM-(P/G)BSA)^4−7^ to more sophisticated alchemical perturbation methods either at equilibrium such as free-energy perturbation (FEP), thermodynamic integration (TI),^8−17^ Bennett acceptance ratio (BAR),^8,12,15,18−23^ and multistate BAR (MBAR)^24−26^ or nonequilibrium like Jarzynski’s equality^27,28^ and Crooks fluctuation theorem.^29^

Perturbation methods require several intermediate lambda states (λ−), in which the ligand can be decoupled, annihilated, or pulled. Theyt use the explicit water definition, and its prediction for relative binding free energies is quite successful. However, they are still at the level of MM energy terms and visit nonphysical intermediate states. All of these methods require a higher computational cost.

Binding free energy (BFE) calculations from single-step end-states are quite attractive since they require only one MD simulation for each protein–ligand (PL) complex system with less computational cost. However, the accuracy is very low due to over-simplifications such as implicit solvent definition in the case of MM-P(G)BSA and molecular mechanics (MM) definition of the Hamiltonian of the system.

Classical force fields, which do not account for electron polarization, charge transfer, and many-body effects, are very limited in accurately defining the protein–ligand interaction. Thus, the system needs to be described at the quantum mechanical (QM) level to include these essential physical effects.^30,31^ There have been several attempts to replace the MM energies with more accurate QM energies. Recently, Fox et al.^30,32^ have introduced a “QM-PBSA” method for eight ligands and improved the MMPBSA energies. One caveat to overcome the prohibitive DFT calculations on thousands of atoms of protein–ligand complexes is to use semiempirical quantum mechanics (SEQM) such as AM1 and SCC–DFTB or QMMM calculations.^33−39^ There are also ongoing efforts to use fragmentation-based QM calculation methods. Most of these studies suffer from no/low sampling from MD simulations.^32,38−52^

Machine learning (ML) techniques have attracted great interest for the past decade due to their successful algorithms to scientific questions including but not limited to chemical reactions,^53,54^ potential energy surfaces,^53,55−57^ forces,^58−60^ atomization energies,^61,62^ and protein–ligand complex scorings.^63^ One of the most promising aspects of the ML techniques is that the trained models can be applied to new systems (transferrable).^53^ In particular, ML potentials with their great capability of learning a multidimensional potential energy surface (PES) at the QM level are promising due to their efficiency and scalability to large systems.^64−66^

Recently, several ML-based neural network potentials (NNPs) such as ANI^67−69^ have been developed using QM energies and forces to predict the potential energy surfaces of small organic compounds. ANI uses modified Behler and Parrinello symmetry functions to construct single-atom atomic environment vectors (AEVs) and could be considered as a quantum accurate force field without requirement of atomic charges. Thus, it only uses atomic symbols and 3D coordinates as an input for the calculations to estimate pairwise atomic interactions. The initial model (ANI-1x) used the PES of nonequilibrium geometries belonging to a total of ∼17.2 million conformations generated from ∼58k small organic compounds composed of C, H, O, and N atoms.^70^ The extended version, ANI-2x, has been shown to predict DFT energies of equilibrium or nonequilibrium conformations of molecules containing C, H, O, N, S, F, and Cl atoms. It has been shown to reproduce the energies at the accuracy of the wb97x/6-31G* level million times faster than the actual QM calculations.^53^

Herein, we introduce a new strategy to estimate the binding free energies of small organic compounds to proteins using end-state MD simulations. The method utilizes ANI-2x neural network potentials and predicts the binding free energies of the protein–ligand (PL) in the linear interaction energy (LIE) formalism. Our results show that the “ANI_LIE” method outperforms current methods for binding free energies. The code is freely available at https://github.com/otayfuroglu/deepQM.

## Theory

### Linear Interaction Energy (LIE)

In the LIE method, only the protein–ligand aqueous complex (PLS) and free ligand in water (LS) are simulated. Assuming that the interactions are additive from the simulations of the protein (P) + ligand (L) + solvent (S) complex system (PLS), it can be derived as
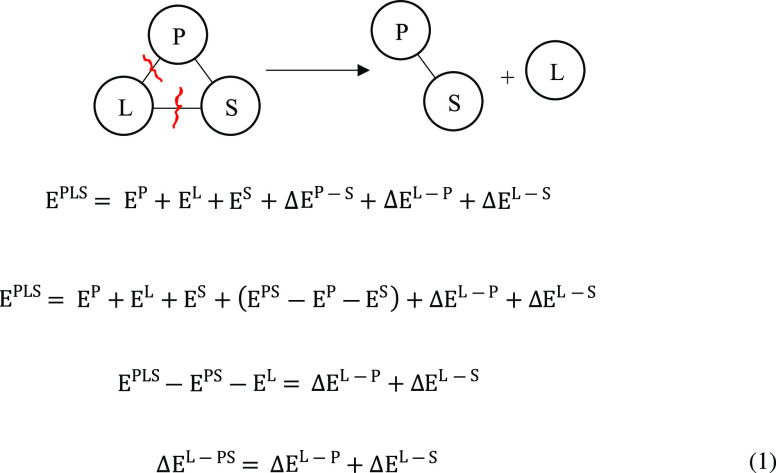
1Thus, the interaction of the ligand with its surroundings can be calculated using the left-hand side of eq 1, which requires defining L as an energy group.
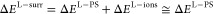
ANI has been trained only for atoms C, O, N, H, Cl, F, and S. Sodium ions cannot be calculated in the current version of ANI-2x. The term Δ*E*^L-ions^ in this equation is the interaction energy of the ligand with ions (which were added to the simulation box to neutralize the protein), and this term can be neglected assuming that the ions will not be nearby the ligands since there are only a few ions in the box and the ligand is mostly buried in the binding pocket (Supporting Information).

The Δ*E*^L-surr^ term can also be calculated from the right-hand side of eq 1 by defining two energy groups, P and L. In that case, the pairwise contributions from each of the separate energy groups of P and L can be extracted and the average interaction between each group is calculated (i.e, Δ*E*^L–P^, Δ*E*^L–S^)
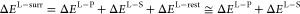
Similarly, from the free ligand in water simulations
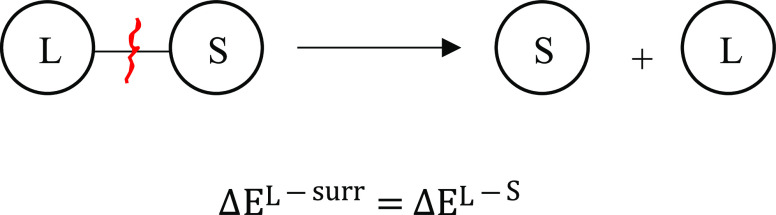
Originating from the linear response approximation (LRA), the binding free-energy change from the bound (PLS) to the unbound state (LS) is given by
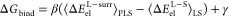
Here, the brackets and their subscripts, ⟨⟩, denote the ensemble average and the components of the simulation, respectively. Δ*E*_el_^L-S^ corresponds to the electrostatic interaction energy term between the ligand (L) and surroundings (solvent, S) from the ligand in solvent simulations.

Thus, the binding free energy can be calculated from
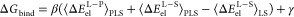
2Including van der Waals terms, the LIE equation becomes
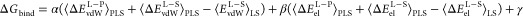
3Here α, β, and γ are empirical parameters determined from linear fitting to the experimental binding energies.

### ANI/D3 Linear Interaction Energy (ANI_LIE)

We explored the success of ANI’s performance on estimation of binding energy using these LIE equations after modifications. Here, we replaced the electrostatic terms with ANI energies and van der Waals terms in the MM energies with Grimme’s dispersion functions (D3) at the wb97x/6-31G* level. In addition, since all of these equations require two simulations (PLS and LS), we have explored either by ignoring solvent terms or borrowing ⟨*E*_ANI_^L–S^⟩_LS_ term from calculations.

In linear response approximation (LRA), “preorganization energy” (POE) is included in ligand–surrounding electrostatic interaction energy. This term is computed from an ensemble in which the partial charges on the ligand atoms are set to zero. On the other hand, the POE term is ignored in a linear interaction energy (LIE) method. With this aspect, ANI follows LIE rather than LRA.

When the solvent is totally ignored and ANI interaction energies are replaced with MM electrostatic energies (similarly, D3 terms replaced with MM van der Waals terms), eqs 2 and 3 become
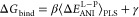
4
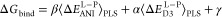
5Although these equations ignore the solvent effects, the source of large noise, which stems from the high mobility of solvent molecules, could be avoided, and thus, more precise (not necessarily more accurate) trends in binding free energies could still be observed.

When the solvent is not ignored, these two equations are updated to
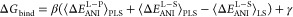
6
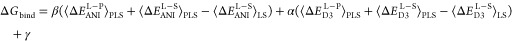
7Here, we generated conformational sampling of protein–ligand (PL), protein (P), and ligand (L) in the presence of explicit water using a single MD simulation (PLS) in the case of solvent ignored calculations. Then, the single-point energies (SPEs) of each MD frame have been calculated by ANI-2x and D3 by stripping out water and ions. For each MD frame, three different energies were calculated: the ligand is complexed with protein, *E*_ANI_^PL^; the bare protein that is extracted from the MD frame, *E*_ANI_^P^, and the bare ligand that is extracted from the MD frame, *E*_ANI_^L^. Similarly, dispersion energy of each component (*E*_D3_^PL^, *E*_D3_^P^, and *E*_D*3*_^L^) has been calculated. When the solvent is included, L–S interaction terms in the PLS MD simulation along with additional simulation of the bare ligand in water (LS) have been considered.

In this study, we used eqs 4–7 to investigate the ANI’s performance of LIE approach (ANI_LIE) in the presence of solvents. Strikingly, we found that the calculations using eq 4 outperform conventional approaches by producing BFEs that strongly correlate with experimental results. The additional terms appearing in eqs (5)-(7) bring subtle improvements to the predictions. Here, our aim is to test the success of the ANI on the interaction energy term; thus, we ignored conformational entropy changes of the ligand and protein upon binding. It has been shown that a single trajectory approach is more precise than the three-trajectory approach due to error cancellation.^30^ Although continuum model definitions such as PBSA could be used in eqs 6 and 7 rather than using explicit solvents, we preferred not to include these terms because the primary aim of this study is to test the success of ANI in the ligand–protein interaction in the LIE approach with explicit waters. It should also be noted that the conformations are already sampled in the presence of explicit waters and ANI-predicted energies even in eqs 4 and 5 belong to equilibrium geometries of aqueous systems (not gas-phase calculations). Therefore, we believe that these conformations inherently reflect the solvent effects at some level. Here, we note that the ANI potentials were used as a postclassical MD simulation to recalculate the MD frames rather than performing the ANI potentials during the MD simulation. Thus, the method is still limited to the classical force field to generate conformational sampling. In addition, the entropic contributions of the free energies are reflected by the width of the energy distributions as with the nature of the LIE approach.

Due to the power of QM-predicted potential energy surfaces, the ANI method can also be applied to ligands that do not have similar structures as opposed to methods such as BAR, FEP, and MMP(G)BSA, in which calculations mostly require ligands with similar scaffolds. We have embedded the end-to-end script to Github for broader access.

## Computational Methods

### System Preparation

For the binding studies, the crystal structures of noncovalent ligands complexed with 3CL^pro^ of SARS-CoV-1, SARS-CoV-2, and HIV-1 proteases, a total of 54 protein–ligand complexes with known IC_50_ values (Supporting Information), were retrieved from the Protein Data Bank (PDB) except for c-Jun N-terminal kinases, which were retrieved from the work by Khalak et al.^71^ Using Gaussian 16 software, the model ligands were first optimized at the B3LYP/6-31G* level and ESP charges were generated at the HF/6-31G* level. Using an Antechamber module in AmberTools 2021, RESP charges and GAFF force field atom types were generated. The Amber2gmx module^72^ from AmberTools 2021 was used to convert amber-type input files to Gromacs.

### MD Simulations

The molecular dynamics simulations were carried out using a Gromacs 2018+ software package^73,74^ with an all-atom model of Amber ff99SB-ILDN^75−77^ force field implemented in Gromacs. The protein–ligand complex (∼6000 atoms) was placed in the center of a dodecahedron box. Each system was solvated in the TIP3P model type water^78^ with a cell margin distance of 10 Å for each dimension. The protein–ligand systems with ∼70,000 atoms were neutralized in 0.15 M NaCl. Classical harmonic motions of all bonds were constrained to their equilibrium values with the LINCS algorithm.

Energy minimization was carried out to a maximum 100 kJ·mol^–1^·nm^–1^ force using a Verlet cutoff scheme. For both long-range electrostatic and van der Waals interactions, a cutoff length of 12 Å was used with the particle mesh Ewald method (PME) (fourth order interpolation).^79^ The neighbor list update frequency was set to 20 ps^–1^. As with our earlier studies,^80−82^ two-step energy minimization and equilibration schemes were used. Each minimization step consisted of up to 50 000 cycles of steepest descent and a subsequent 50 000 cycles of l-bfgs integrators.

After minimization, each system was equilibrated within three steps using Langevin dynamics. The first step consisted of a 1 ns of NVT ensemble. The protein–ligand and the rest of the system were defined as two temperature groups at 310 K. The next step consisted of NPT ensembles, in which the systems were equilibrated to 1 atm pressure by Berendsen for 200 ps and followed by Parrinello-Rahman isotropic pressure coupling for 1 ns to a reference pressure of 1 atm. When systems reached to equilibrium, an MD simulation of 10 ns was carried out at the NPT ensemble.

### Free Energy Calculations

Simulations for alchemical free energy calculations were carried out using a Gromacs 2018+ software package^73,74^ with the all-atom model of Amber ff99SB-ILDN^75−77^ force field implemented in Gromacs. For the alchemical free energy calculations, 10 equal decoupling steps (i.e., decoupling the ligand from the environment) of each Coulombic and van der Waals interaction (Δλ = 0.1, total 21 λ-windows) were used. For the calculations of the protein–ligand complex, a separate decoupling of the ligand from the ligand+water system with the same parameters was performed and subtracted from the decoupling of the ligand from the protein + ligand + water complex system.^80^ An *Alchemicalanalysis* module from the *pymbar* library^83^ was used to print alchemical free energies.

MM-PB(GB)SA calculations were performed using gmx_MMPBSA script byAmberTools 2021 v4. For the calculations of protein–ligand complexes, we used the λ = 0 trajectories of protein+ligand+water complexes from alchemical free energy calculations. Only the protein + ligand complex part from the trajectories was extracted and default parameters of implicit water with ε = 80 and solute with ε = 2 were used. Nonpolar solvation parts were separated into dispersion and cavity terms, which is defined by solvent accessible surface area from molecular volume.

ANI and D3 calculations were performed using our custom-built python scripts deepQM as with our previous study for LS solvation free energies (data unpublished). Since it corresponds to a fully interacting state, we used a trajectory of the first window (λ_coul_ = 0; λ_vdw_ = 0) from the alchemical MD simulations for QM accuracy calculations using ANI and Gaussian 16 software (G16). For G16 calculations at the wb97x/6-31G* level, it is computationally too expensive to carry out the entire protein–ligand system. Therefore, we had to truncate the residues around the ligand by 4.0 Å (nearly 900 atoms). Gromacs software was utilized to index energy groups, remove PBC, order the nearest waters around the PL complex, and extract structures from trajectories for ANI calculations while PyMol was used to truncate the nearest residues around the ligand for the QM calculations at Gaussian 16 software.

## Results and Discussion

### Workflow

deepQM is written in Python 3.9 (details of the functionality and different modules of deepQM are discussed elsewhere) and uses ASE libraries to calculate ANI and DFT-D3 single-point energies of the given A–B complex structure (e.g, A = protein, P; B = ligand, L). As an end-to-end process with the DFT accuracy, binding free energy is predicted from the PL/LS complex, free protein, and ligand structures, all of which are extracted from the trajectories of classical MD simulations of the protein–ligand complex system in water.

**Scheme 1:**
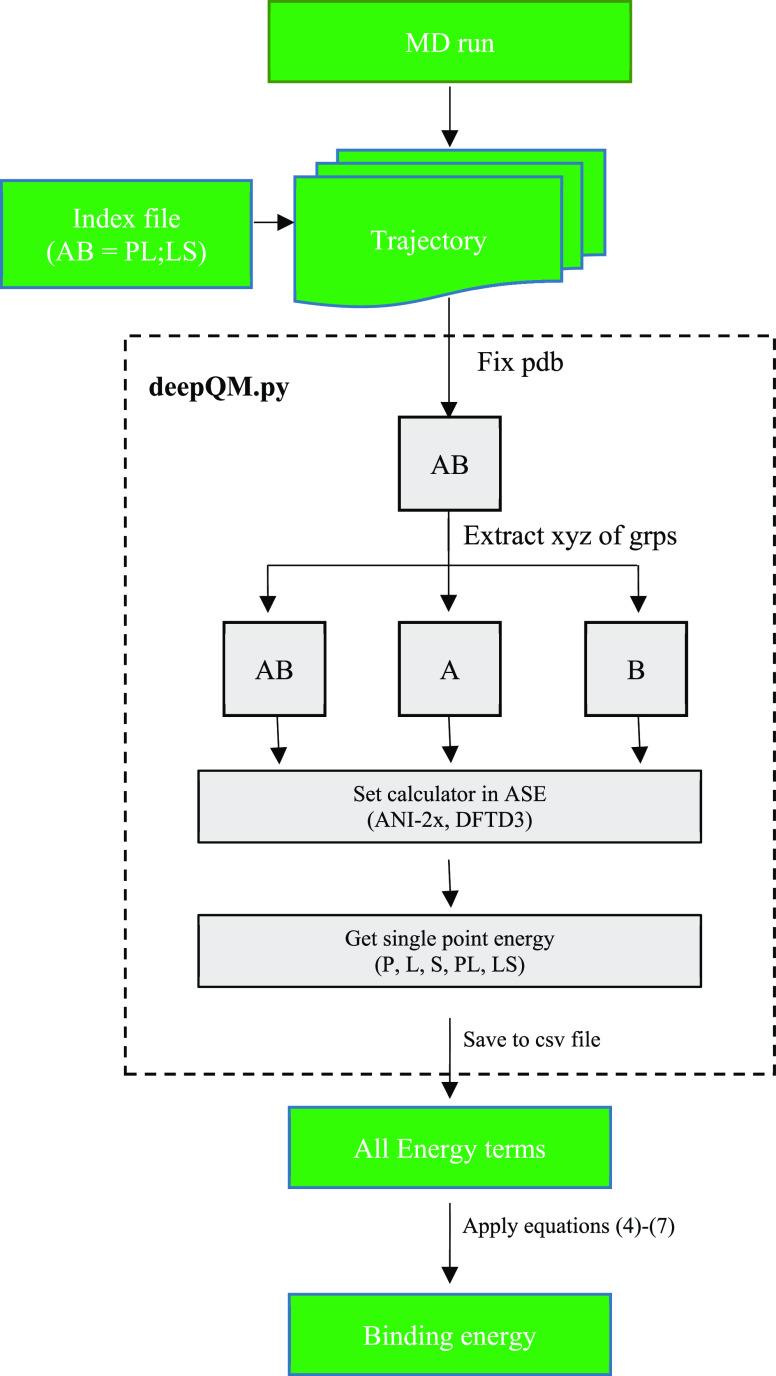
deepQM General Workflow for Performing End-State Binding Free Energy Calculations The first step consists of extracting individual groups and converting to xyz file format. The second step is the calculator. Single or multicalculators can be selected. Finally, the results were printed and combined to predict binding free energy.

The workflow for the prediction of protein–ligand binding free energy from multiframes of an MD simulation is as follows: First, the atomic indices of the protein and ligand are read from the index groups of the index file and extracted into three separate xyz coordinates (PL, P, L or LS, L, S) after preprocessing the pdb files to make compatible for deepQM (Scheme 1). Next, ANI and DFT-D3 calculators are imported and set from the ASE platform, and then, each group is read by both calculators to predict total single-point energy (SPE) and D3 dispersion energy, respectively. Next, the interaction energy between the protein and ligand is written to data files for each frame in both methods. In the final step, binding free energy is estimated using the equations discussed in the Computational Methods section. In principle, it can work with any MD simulation packages upon converting trajectories to separate pdb files that contain the protein–ligand–solvent complex and defining energy groups. Index file format must be Gromacs type.

### ANI Results

#### Can ANI Reproduce DFT Interaction Energies?

Since ANI has been trained using atomic environments, it could still be scalable to the DFT calculations for larger systems such as protein–ligand interactions. It should be noted that DFT single-point energies of the protein and protein–ligand complex systems are in the order of hundreds of thousands of eVs, whereas the binding energy is only a few eVs. Therefore, it is important to have the total energies converged for accurate binding free energy calculations. Table 1 shows the absolute energies of truncated protein+ligand complex systems (HIV protease, PDB ID = 5IVS) calculated by ANI and G16 at the wb97x/6-31G* level. There are 55 residues around the ligand in the truncated protein with 920 atoms (Figure S1). ANI’s prediction on ligand’s energy is excellent. However, the ANI predicted absolute value of the protein–ligand complex deviates by ∼2 eV from that of DFT calculations. However, this energy is just a bias and canceled out by subtracting the energy of the protein in the interaction energy term. This bias cancellation will also occur on extended (untruncated) systems (i.e., a full protein + ligand complex). Therefore, the ANI-predicted interaction energies are in reasonable agreement with G16 calculations, with a shift by only ∼0.252 eV (5.812 kcal/mol). This value can be minimized so as to converge by increasing the number of frames sampled by the MD simulation and increasing simulation time. It should also be noted that it has been trained for atomic environments with a cutoff (5.1 Å for radial and 4.5 Å for angular chemical environments), which limits its calculation to short-range interactions.

**Table 1:** Absolute Energies of Truncated Protein + Ligand Systems (HIV Protease, PDB ID = 5IVS) from MD Simulations (in eV)^a^

	G16				ANI				
frame	complex PL	receptor P	ligand L	Δ*E*_int_	complex PL	receptor P	ligand L	Δ*E*_int_	abs. err.
0	–601 409.137	–543 088.071	–58 316.912	–4.154	–601 407.549	–543 086.919	–58 316.653	–3.977	0.177
1	–611 338.434	–553 017.357	–58 317.024	–4.053	–611 337.259	–553 016.927	–58 316.904	–3.428	0.625
2	–611 343.896	–553 023.284	–58 316.737	–3.876	–611 342.856	–553 022.874	–58 316.543	–3.439	0.436
3	–611 338.693	–553 021.427	–58 316.102	–1.164	–611 337.181	–553 020.251	–58 315.918	–1.012	0.152
⋮									
100	–611 340.901	–553 023.152	–58 315.756	–1.992	–611 340.670	–553 023.116	–58 315.703	–1.878	0.115
mean	–611 366.228	–553 047.220	–58 316.584	–2.424	–611 368.035	–553 046.484	–58 319.368	–2.183	0.252

a For the complete 100 frames, refer to the Supporting Information.

**Figure 1:**
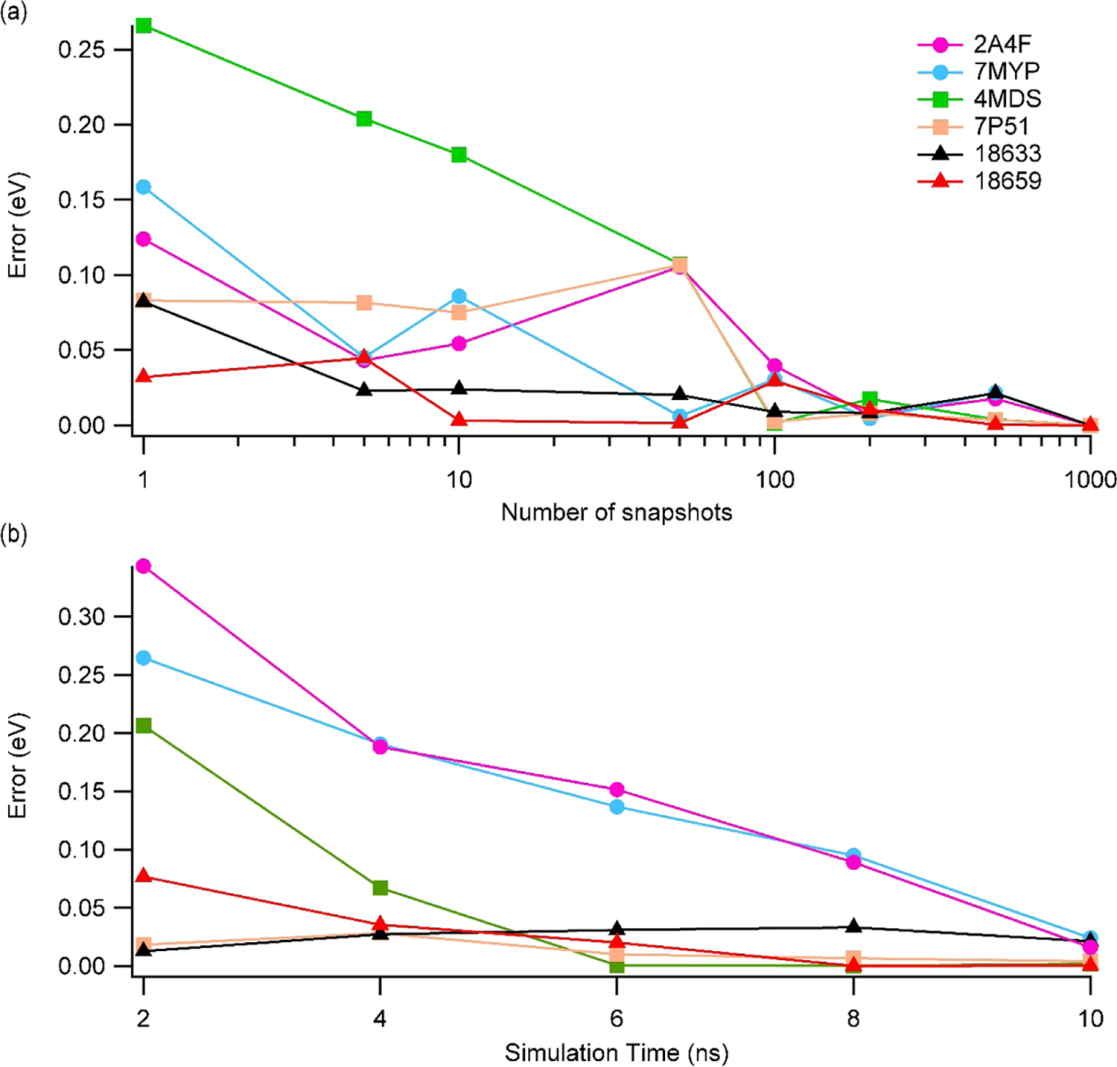
Absolute deviations of the average P–L interaction energies for selected complexes calculated by ANI, considering 1000 frames and 10 ns simulations as the converged value (a) as a function of the number of frames to average and (b) as a function of simulation time using only 100 frames in each time interval.

To assess the convergence of the total energy, we have extracted snapshots from the trajectories of 10 ns MD simulations by retrieving the first frames in different time intervals (e.g, 10 ps for 1000 frames). Figure 1a shows the convergence of the average interaction energy when more snapshots are averaged, assuming 1000 frames in 10 ns MD simulations as the fully converged energy. The data suggests that using 100 frames in the average calculations is sufficient to have a convergence to a maximum of 0.04 eV (1.1 kcal/mol). This error drops to below 0.02 eV (0.46 kcal/mol) when 200 frames are used.

We have also investigated how MD simulation time affects the interaction energy (Figure 1b). It suggests that the simulation time does have more impact on the absolute errors and an 8 ns of simulation time can bring a maximum error of 0.09 eV (2.5 kcal/mol). This error reduces to below 0.5 kcal/mol in 10 ns simulations.

One of the most important features of the ANI calculations is that they can be run on GPU or CPU and either construction can be run in parallel. The computation time is million times faster than DFT calculations. On a 4x NVIDIA Tesla V100 construction, the 1000 frames of the P–L and L–S complexes can be calculated within hours if not minutes. A single frame in our truncated P–L structures with 55 residues and 920 atoms could take several hours at the wb97x/6-31G* level.

#### Can the Electrostatic Interaction Term Be Replaced by ANI?

**Figure 2:**
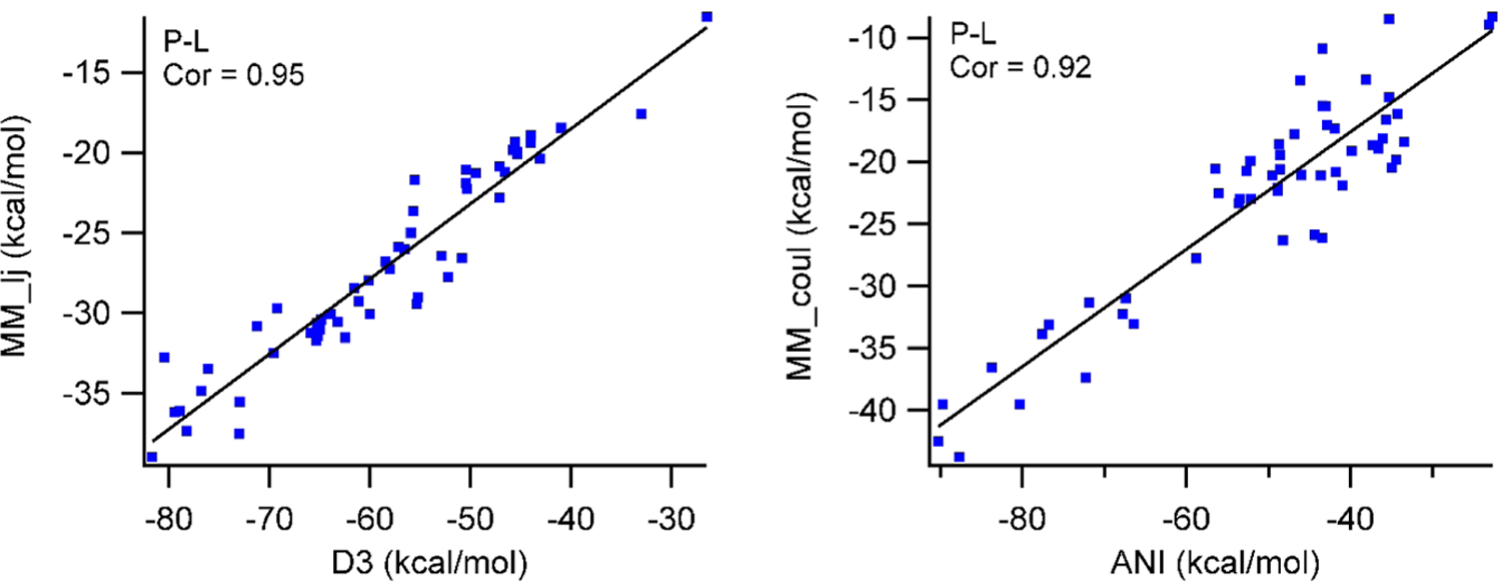
ANI-predicted average interaction energies and classical electrostatic interaction energies between the protein and ligand from PLS simulations are correlated while the energies from Grimme’s D3 functions are correlated to van der Waals interactions.

The ANI and D3 energy terms are correlated and can be replaced with Coulombic and van der Waals terms, respectively. As both van der Waals interaction terms in MM and Grimme’s D3 functions originate from Lennard-Jones potentials, it would be trivial to correlate these two terms. Thus, the original LIE equation can easily be adopted by replacing D3 energies with MM-based van der Waals terms and by replacing MM-based electrostatic interaction energy terms with ANI-predicted interaction energies (Figure 2). ANI also shows great correlation to electrostatic terms, which allows us for the modification of the LIE equation.

Since ANI and MM energy terms are strongly correlated, one should expect LIE calculation by both methods to follow similar trends with respect to experimental free energies. Surprisingly, the correlation of ANI_LIE values to experimental free energies is greater by ∼15% than that of MM_LIE.

#### BFE by Ignoring LS Interaction

**Figure 3:**
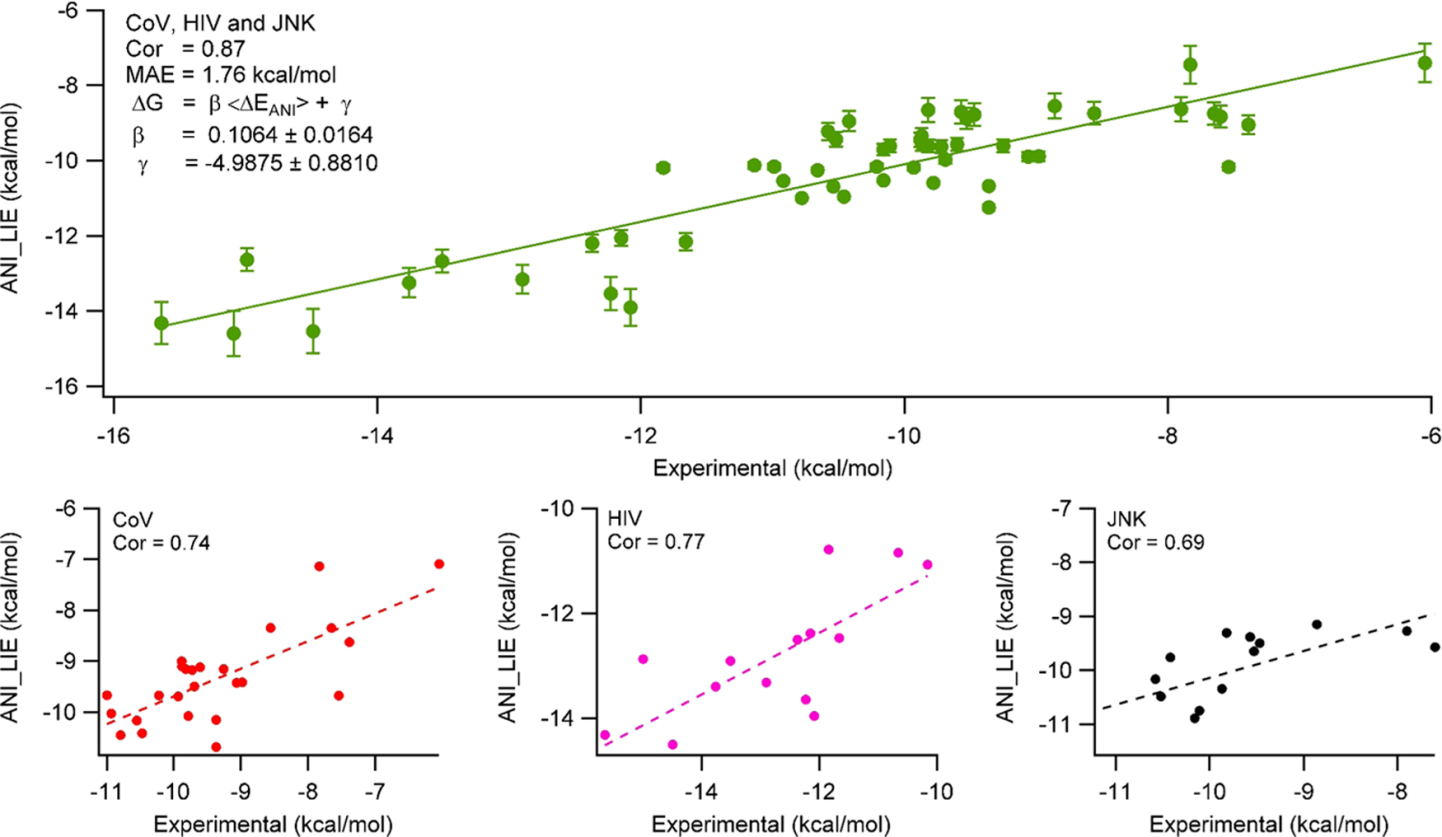
ANI interaction energies calculated from eq 4 with β = 0.10639 ± 0.0164 and γ = −4.9875 ± 0.881 using average of 100 snapshots of 10 ns MD simulations for 54 protein–ligand complexes, showing 0.87 correlation to the experimental values. The bar lines show uncertainties. The bottom plots show the fits of individual protein families as CoV, HIV-1, and JNK-1.

Using the simplest modification of the LIE formula (i.e., eq 4), we have calculated the ANI interaction energies. The coefficients were determined from the fit to the experimental values (Figure 3). It should be emphasized that there are three different protein families with a total of 54 complexes and the ligands do not necessarily have similar scaffolds. We observed excellent agreement (*R* = 0.87) between the experimental and ANI/LIE-predicted absolute binding free energy values. The mean absolute error (MAE) is just 1.76 kcal/mol. Similar to LIE calculations using only ANI potentials, we also investigated the effect of the inclusion of D3 terms in the prediction using eq 5. Although the correlation coefficient was not improved significantly, the MAE value decreased to 0.76 kcal/mol (Figure S2).

#### BFE by Including LS Interaction

After showing the success of ANI’s prediction of interaction energies and binding free energies in the absence of solvents, we have also attempted to calculate the binding free energies using the most extensive LIE formalism in which the solvent effects are also included. The solvent requires a second simulation of ligand in water (LS) along with the contribution of L–S interactions in the PLS simulations as explained in the Theory section.

In principle, ligand–solvent interactions can be computed with implicit definitions as in the case of PBSA, GBSA, or any other continuum models. For instance, a recent work by Fox et al. introduced the so-called “ONETEP” program, in which they coupled the DFT energies with PBSA to calculate QM-PBSA energies.^30^ However, here, we attempted the L–S interaction energies using explicit waters in the system sticking with the original formalism of the LIE method.

**Figure 4:**
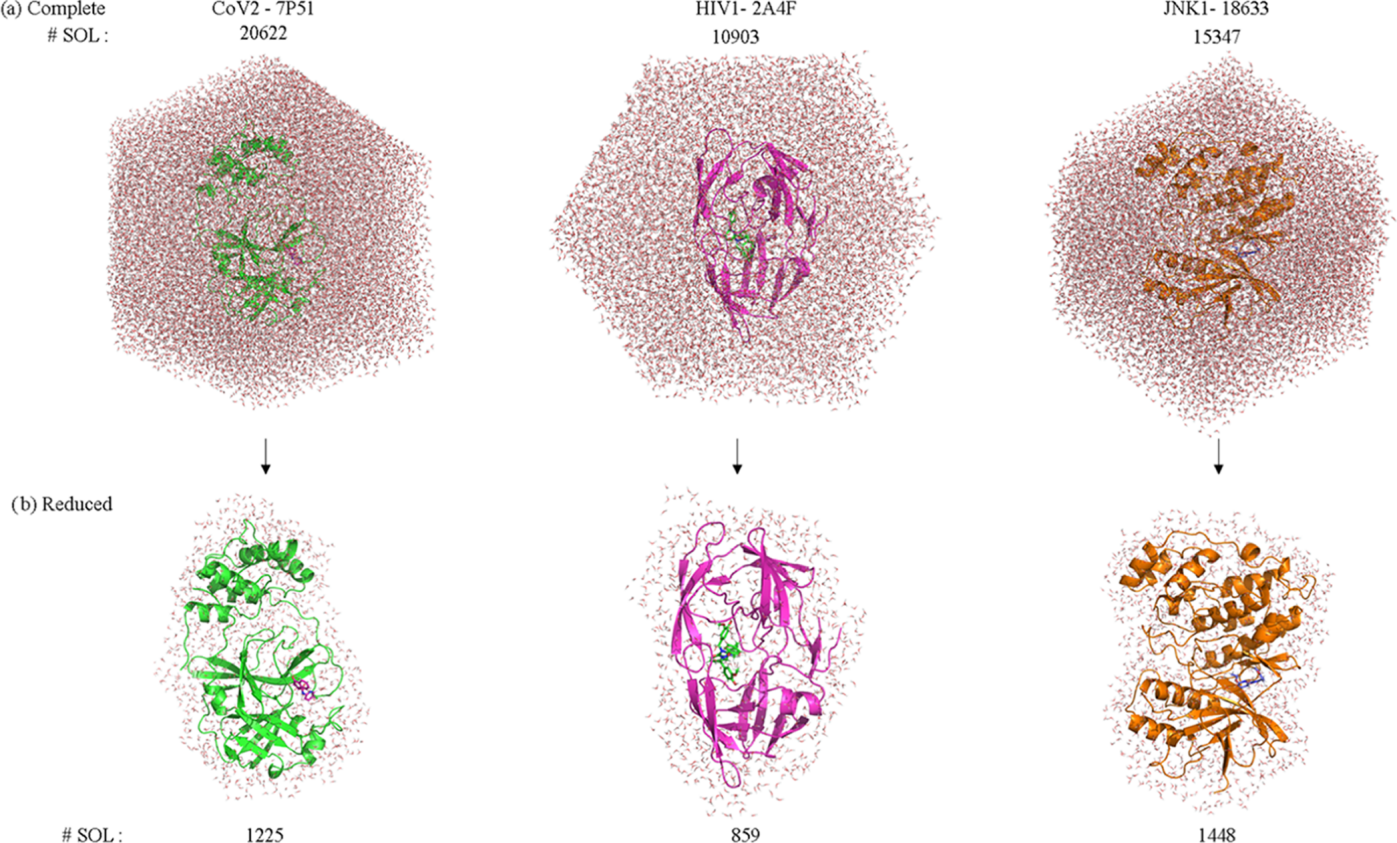
Illustration for the representative structures in protein + ligand + complete/reduced water (PLS) systems. The LIE calculations were performed using reduced water to ease memory cost of the computations. The results are almost identical in both cases (Table S1).

Although ANI enables us to calculate such large protein+ligand+water (PLS) systems, due to memory limitations, we could only perform L–S interaction energies after reducing the PLS systems so as to include waters by only 4 Å around the PL complex. We achieved this by ordering the water in the trajectories and extracting the nearest solvents in the PLS system. Figure 4 shows examples of reduced vs complete water in PLS systems for each protein family. Since the ligand is mostly buried in the protein, there are only a limited number of solvents around the ligand in the bound state. Thus, the effect of the solvents that are beyond 4 Å should not affect the calculations. Indeed, one can see the comparison of MM energy terms (Coulombic, van der Waals) along with MM_LIE calculations by taking only reduced water in the system rather than the complete water (Table S1).

**Figure 5:**
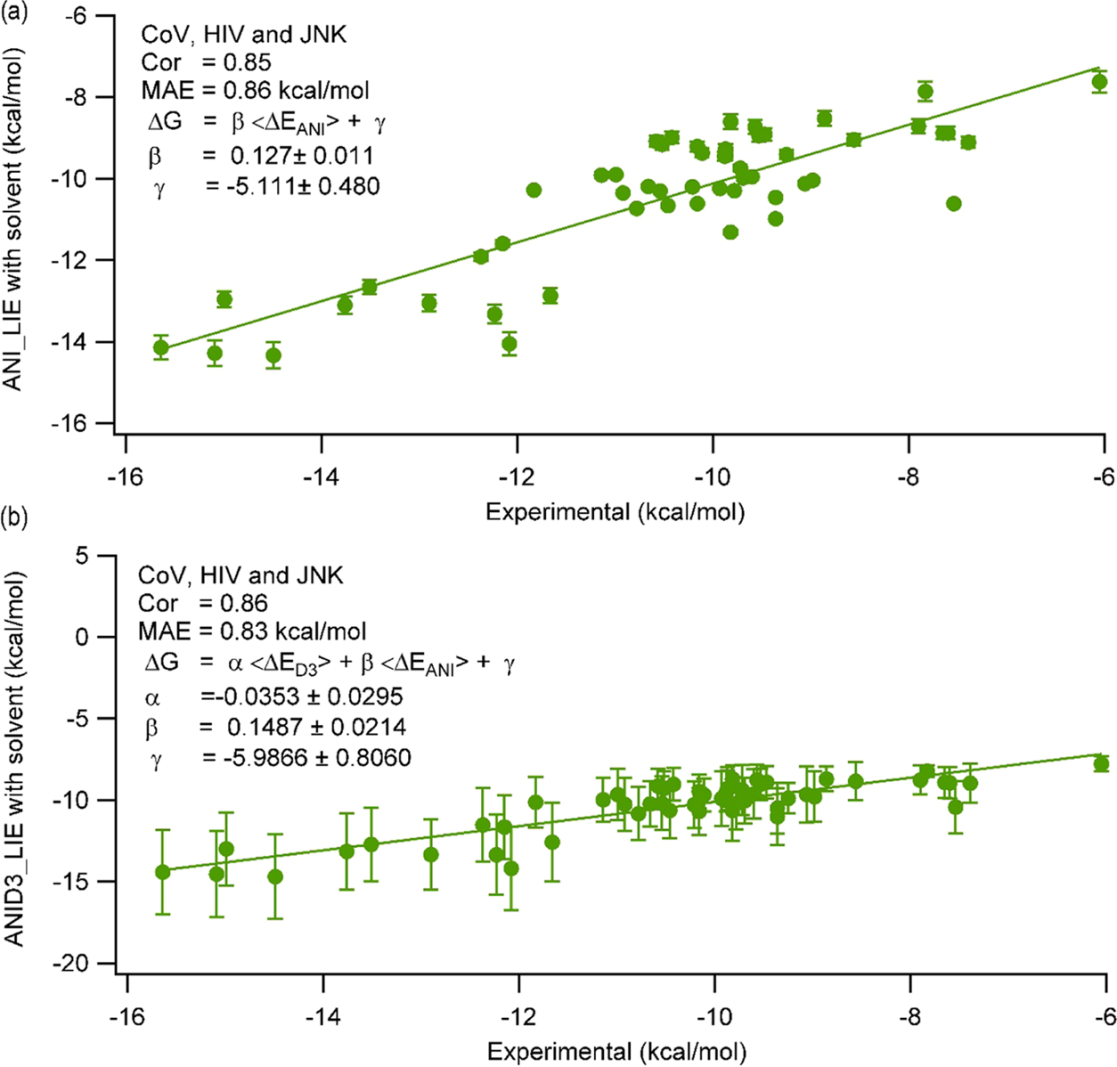
LIE calculated by ANI and ANID3 using (a) eq 6 and (b) eq 7, in which the solvent effects around the ligand were included in the calculations.

By applying eqs 6 and 7, we could successfully produce the absolute binding free energies based on ANI/D3 interactions. Figure 5 shows that the solvent included ANI and ANID3 LIE calculations. We have summarized the correlation coefficients for different approaches in Table S2.

### Benchmarking with Other Methods

#### Binding Free Energies from the Classical LIE Approach

In addition to the ANI method introduced here, we also performed calculations from several other methods for the studied protein–ligand complexes. The very first method was the classical LIE method, in which Coulombic and van der Waals interactions at the level of molecular mechanics were computed and fit to the experimental binding free energies to determine the coefficients.

The default parameters of LIE in Gromacs software use α = 0.181, β = 0.5, and γ = 0 and yield very low correlation to the experimental values. The β coefficient for the electrostatic interactions was reported to have several values according to the charged vs uncharged ligands. Studying 18 PL complexes, Hansson et al.^84^ concluded that β = 0.5 is a good approximation for charged ligands whereas lower values such as 0.43, 0.37, and 0.33 for neutral molecules with 0, 1, or >1 hydroxyl groups, respectively.^85^ Since then, it has been used with care by fitting to the new sets of protein–ligand complexes.^86−91^

**Figure 6:**
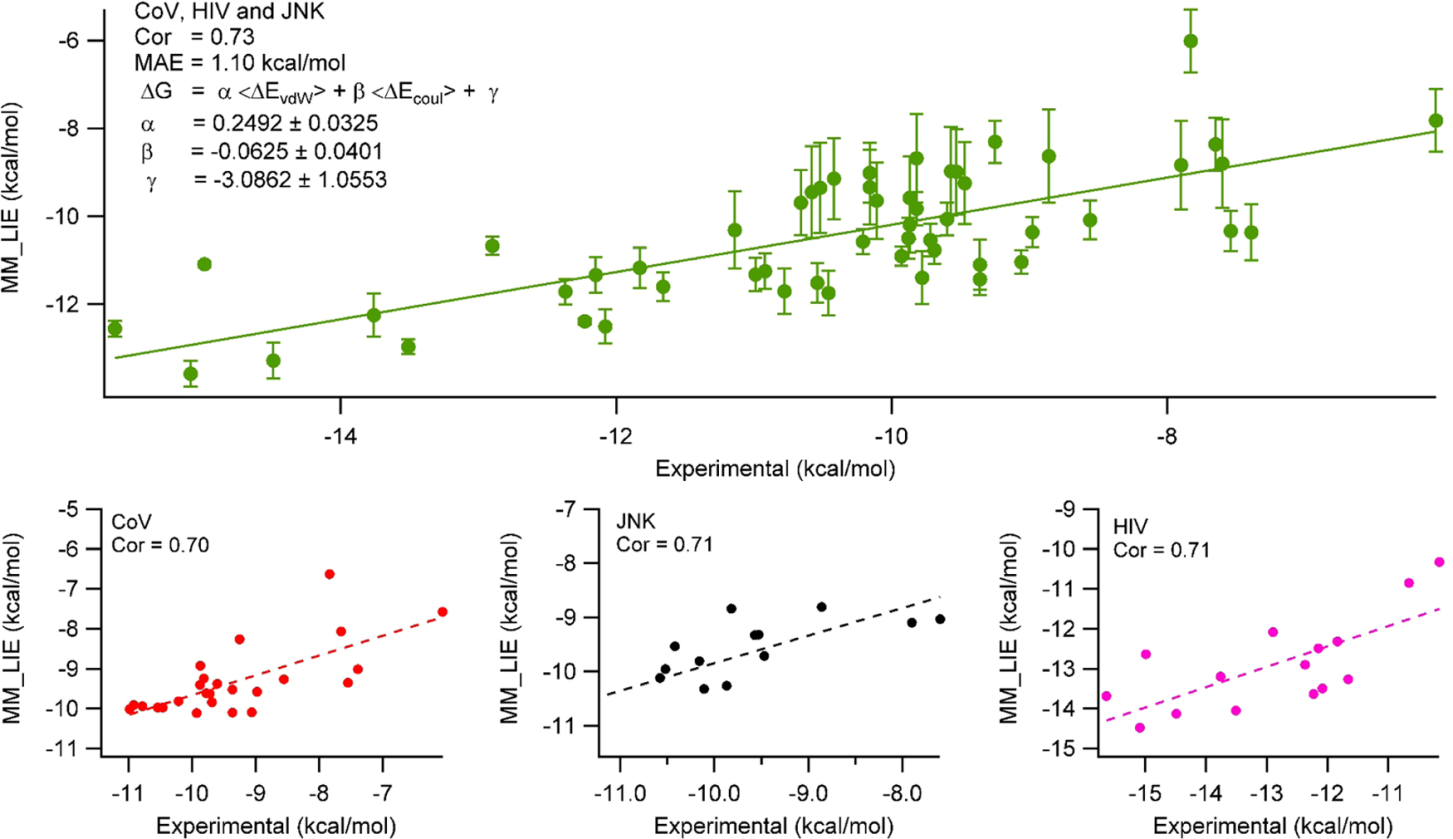
LIE calculated by MM electrostatic and van der Waals interactions using eq 3. The parameters were determined from the fit. The bottom plots show the correlation in each individual protein family.

With the default parameters (α = 0.181, β = 0.5, and γ = 0), we have observed very low correlation between the experimental and MM_LIE prediction (Figure S3). On the other hand, when new parameters are assigned from our fit, we observe 0.70–0.73 correlation with α = 0.25, β = −0.06, and γ = −3.09 (Figure 6). Here, all of the ligands studied are neutral and we did not classify them according to the presence of polar groups like hydroxyl groups. Note that MM_LIE is still quite successful (*R* = 0.70–0.73), and it is clear that ANI_LIE (*R* = 0.85–0.87) is much better in reproducing experimental values.

In addition to the parameters calculated from all 54 data points of the dataset for both MM_LIE and ANID3_LIE, we have also analyzed the data by splitting training (90%, 48 points) and test sets (10%, 6 points). We have done this by randomly selecting 100 different train and test sets. The coefficients were produced from the training sets and applied on test sets. The coefficients generated from random sampling and whole dataset are in a good agreement. We have reported the predicted values for the coefficients and their RMSE values on training and test sets (in the Supporting Information and Table S2).

We have also performed MMPBSA and MMGBSA calculations as a comparison to ANI. The computational cost of these two methods is very similar to ANI since they are also end-state methods applied on bound-state (PLS) classical MD simulations. We should note that these methods do not use empirical fitting coefficients in the calculations. However, their success of predicting absolute binding energies can become very poor according to the system and they are mostly used to predict the relative binding free energies. In particular, when the ligands have similar scaffolds, they can accurately predict relative binding free energies. Similarly, when the same ligand’s binding to a protein and its mutations were compared, they can be quite successful. We have tested the performance of both MMPBSA and MMGBSA methods for the protein–ligand complexes studied here.

Due to the fact that protein families of the ligands are very different, we could only observe somewhat meaningful correlations when the protein families were analyzed separately in the case of MMPBSA. When all proteins were treated with the same preassigned values of MMPBSA, we observed very poor correlation to the experimental values (Figure S4).

Surprisingly, MMGBSA showed a somewhat similar correlation (*R* = 0.81) with classical LIE to the experiments even when all 54 protein–ligand complexes were analyzed together (Figure S5). This is still less than the ANI-predicted values of *R* = 0.87–0.88.

We have also explored the binding energies by means of alchemical methods using a 10 λ decoupling window for electrostatic interaction and 10 λ for van der Waals interactions. During decoupling, no restraint was applied on the ligands; 10 ns from each window brings 20-fold computational cost to the simulations, whereas it still has a bare correlation to the experimental binding free energies (Figure S6). These low correlations may be due to the limited number of windows.

## Summary and Conclusions

Here, we applied ANI-ML potentials, trained at the wb97x/6-31G* level, to calculate the interaction energies of protein–ligand and ligand–solvent pairs. The results show that the predicted interaction energies even when the solvent is totally ignored are highly correlated with experimental values. By modification of the LIE method, we have assigned coefficients for the ANI_LIE method. The ANI_LIE method outperforms conventional methods like LIE, BAR, and MMP(G)BSA in terms of accuracy and is comparable to MMP(B)SA in terms of computational cost. Our preassigned parameters determined for the method can be applied on any protein–ligand complex systems even when the protein(s)/ligand(s) have different scaffolds.

## Supporting Information Available

Supplementary figures of truncated protein + ligand in the DFT calculations, ANID3_LIE results, MMP(G)BSA and BAR results and table of the classical LIE when the complete/reduced solvents considered (PDF)
Raw data of all outputs (XLSX)

## Notes

The ANI_LIE method using deepQM along with tutorials and descriptions can be accessed via [https://github.com/otayfuroglu/deepMOF.git]. Input and output files from simulations can be accessed via: https://doi.org/10.5281/zenodo.6538001.
