# Supplementary material for: Accurate Binding Free Energy Method from End-State MD Simulations
Source: J Chem Inf Model. 2022 Aug 16;62(17):4095–106. doi: 10.1021/acs.jcim.2c00601 (PMC9472276; doi:10.1021/acs.jcim.2c00601)
Supplement: Supplementary file 1 — ci2c00601_si_001.pdf [file ci2c00601_si_001.pdf]

Supporting Information

An accurate binding free energy method from end-state MD simulations

Ebru Akkus, Omer Tayfuroglu, Muslum Yildiz, and Abdulkadir Kocak[1]

Department of Chemistry, Gebze Technical University, 41400, Kocaeli/Turkey

[1] Corresponding to: Abdulkadir Kocak, Email: kocak@gtu.edu.tr, Phone: +902626053083

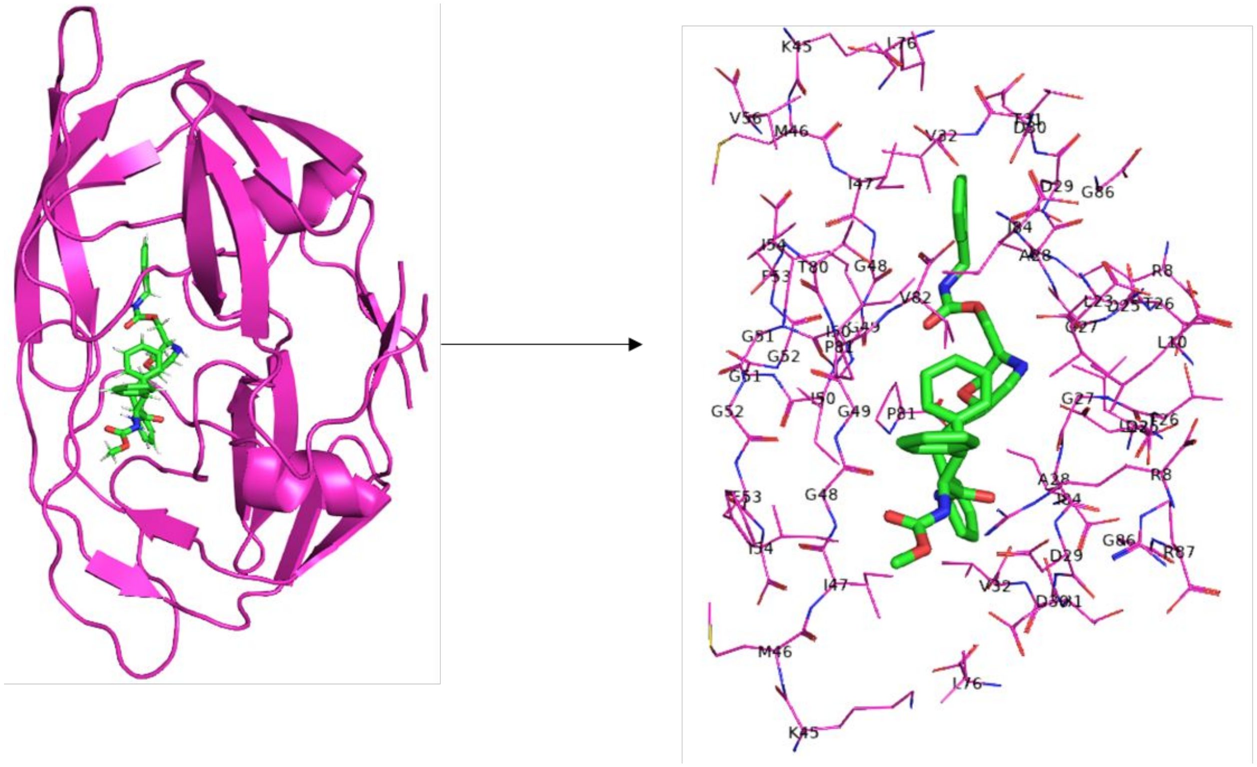

CHAIN	RESIDUES
A	8<br>23<br>25-32<br>45-54<br>76<br>81<br>84<br>86-87
B	8<br>10<br>23<br>25-32<br>45-54<br>56<br>76<br>80-82<br>84<br>86

Figure S1: Truncated protein-ligand system (HIV protease, PDB id=5IVS) in the DFT calculation as a comparison to ANI, in which all the P-L system can also be calculated. All the residues were saturated with H atoms to make the structure neutral. All H atoms were ommitted from structuress for better view. The table shows which residues included in the truncated protein.

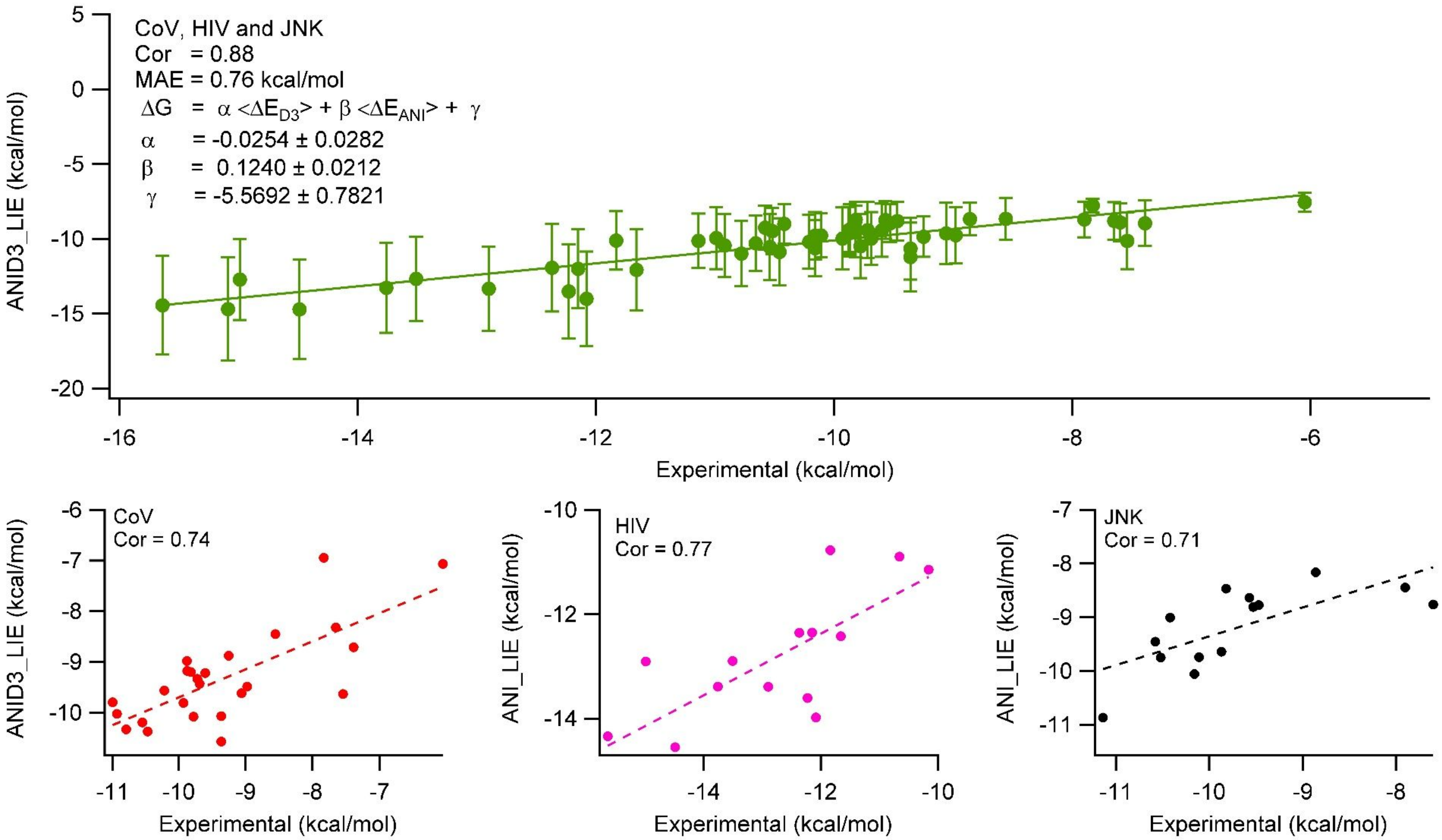

Figure S2: ANID3 interaction energies calculated from the equation (5) with α=-0.0254±0.0282, β= 0.1240 ± 0.0212 and γ=-5.5692 ± 0.7821 using average of 1000 snapshots of 10 ns MD simulation for 54 protein ligand complexes, showing 0.88 correlation to the experimental values. The bar lines show the mean absolute error. The bottom plots show the fits of individual protein families as CoV, HIV-1 and JNK-1.

Table S1: MM energy terms and LIE for complete vs reduced water, showing reducing system hardly affects the results.

	Complete water		α=0.181; β=0.5	Reduced water		α=0.181; β=0.5			
PDB ID	L-rest_ coul (kcal/mol)	L-rest_LJ (kcal/mol)	LIE_whole systemt (kcal/mol)	L-rest_coul (kcal/mol)	L-rest_LJ (kcal/mol)	LIE reduced water only 4Awaters (kcal/mol)	Diff_coul	Diff_lj	Diff_LIE
2GZ7	-10.76	-11.29	-3.23	-9.92	-9.94	-2.57	-0.84	-1.35	-0.67
2GZ8	-18.02	-17.36	-2.09	-18.21	-14.33	-1.63	0.19	-3.03	-0.45
3V3M	-13.71	-11.04	-3.07	-10.51	-10.38	-1.35	-3.20	-0.67	-1.72
4MDS	-24.22	-17.62	0.54	-24.48	-15.78	0.75	0.26	-1.85	-0.20
4YOJ	-19.46	-10.19	-3.18	-19.61	-8.74	-2.99	0.15	-1.46	-0.19
7L10	-7.89	-13.27	-1.12	-8.59	-11.95	-1.23	0.69	-1.31	0.11
7L11	-4.85	-12.07	-2.11	-5.40	-11.01	-2.19	0.55	-1.06	0.08
7L12	-20.46	-12.98	1.01	-20.92	-11.38	1.07	0.46	-1.60	-0.06
7L13	-24.10	-13.36	-1.51	-24.65	-11.87	-1.51	0.55	-1.50	0.01
7L14	-4.96	-13.68	-2.45	-5.49	-12.44	-2.49	0.52	-1.23	0.04
7LMF	-12.31	-11.89	-3.15	-12.69	-10.76	-3.13	0.38	-1.13	-0.02
7LMG	-14.46	-11.42	-2.65	-14.69	-10.38	-2.57	0.23	-1.04	-0.07
7LMH	-15.27	-13.71	-1.78	-15.72	-12.38	-1.77	0.45	-1.34	-0.01
7LMI	-14.99	-11.89	-1.12	-15.26	-10.82	-1.06	0.28	-1.08	-0.06
7M8M	-25.97	-11.67	-2.64	-26.39	-10.35	-2.61	0.42	-1.33	-0.03
7M8N	-27.85	-13.39	-3.78	-28.22	-11.67	-3.65	0.36	-1.72	-0.13
7M8O	-19.07	-13.19	-0.79	-19.16	-11.69	-0.56	0.09	-1.50	-0.23
7M8P	-23.13	-14.29	-1.10	-23.96	-12.59	-1.20	0.82	-1.70	0.10
7M8X	-10.86	-13.16	-2.46	-11.54	-11.86	-2.56	0.68	-1.30	0.11
7M8Y	-25.35	-16.43	0.47	-25.82	-14.49	0.58	0.48	-1.95	-0.11
7M8Z	-28.66	-12.70	0.32	-28.94	-10.92	0.50	0.28	-1.78	-0.18
7M90	-33.05	-14.38	-1.91	-33.09	-12.64	-1.61	0.04	-1.73	-0.29
7M91	-18.63	-10.96	0.34	-18.98	-9.99	0.34	0.35	-0.97	0.00
7N44	-22.70	-12.97	0.20	-23.08	-11.48	0.28	0.38	-1.50	-0.08
7P51	-14.64	-9.94	-0.38	-14.95	-9.07	-0.38	0.31	-0.86	0.00
2A4F	-23.79	-9.82	0.20	-23.82	-9.01	0.33	0.02	-0.81	-0.13
3R0W	-55.87	-15.94	1.13	-55.73	-15.06	1.35	-0.13	-0.88	-0.22
3R0Y	-38.54	-16.56	-0.95	-38.43	-15.59	-0.72	-0.12	-0.98	-0.23
3SPK	-8.94	-8.21	-0.63	-8.83	-7.77	-0.49	-0.11	-0.44	-0.14
4KB9	-19.53	-6.86	-2.31	-19.52	-6.38	-2.22	-0.01	-0.49	-0.09
5DGW	-16.30	-10.96	0.39	-16.40	-10.19	0.48	0.10	-0.76	-0.09
5IVS	-31.12	-15.71	-9.37	-31.25	-14.20	-9.16	0.13	-1.51	-0.21
5IVT	-26.26	-10.23	-4.20	-26.14	-8.98	-3.91	-0.12	-1.25	-0.29
5TYS	-31.76	-11.71	-3.09	-31.50	-10.64	-2.76	-0.26	-1.07	-0.33
5ULT	-18.51	-9.58	-1.06	-18.77	-9.03	-1.10	0.26	-0.55	0.03
5UOV	-36.78	-25.32	-0.16	-37.10	-22.58	0.17	0.32	-2.73	-0.33
6CDJ	-32.87	-17.31	-6.78	-33.08	-15.98	-6.64	0.20	-1.33	-0.14
6CDL	-11.60	-13.63	-0.90	-11.72	-12.82	-0.82	0.12	-0.80	-0.09
6VOE	-31.69	-13.75	-7.78	-21.30	-13.81	-2.60	-10.38	0.06	-5.18
7MYP	-17.19	-9.22	-4.31	-17.19	-8.82	-4.24	0.00	-0.41	-0.07
18624	-6.77	-8.06	4.23	-6.96	-7.39	4.25	0.19	-0.67	-0.03
18629	-9.01	-8.22	3.77	-9.16	-7.50	3.83	0.15	-0.72	-0.05
18630	-6.19	-8.41	3.49	-6.49	-7.62	3.48	0.30	-0.79	0.01
18631	-4.78	-8.01	3.61	-5.02	-7.27	3.63	0.24	-0.74	-0.01

18632	-10.81	-8.45	3.13	-10.98	-7.68	3.18	0.17	-0.76	-0.06
18633	-6.03	-9.12	3.86	-6.16	-8.26	3.95	0.13	-0.86	-0.09
18634	-9.23	-9.02	2.98	-9.39	-8.17	3.05	0.16	-0.84	-0.07
18635	-5.55	-8.80	3.63	-5.69	-7.95	3.71	0.14	-0.84	-0.08
18637	-19.97	-11.21	4.25	-19.75	-9.80	4.61	-0.21	-1.40	-0.36
18638	-23.61	-10.50	3.63	-22.68	-9.04	4.36	-0.93	-1.46	-0.73
18639	-10.28	-10.38	2.11	-10.12	-9.19	2.40	-0.16	-1.19	-0.29
18652	-17.17	-10.36	3.05	-16.87	-9.01	3.45	-0.30	-1.34	-0.39
18658	-17.49	-9.25	2.63	-17.49	-8.23	2.81	0.00	-1.02	-0.18
18659	-11.53	-10.09	3.48	-11.73	-9.10	3.56	0.20	-0.99	-0.08

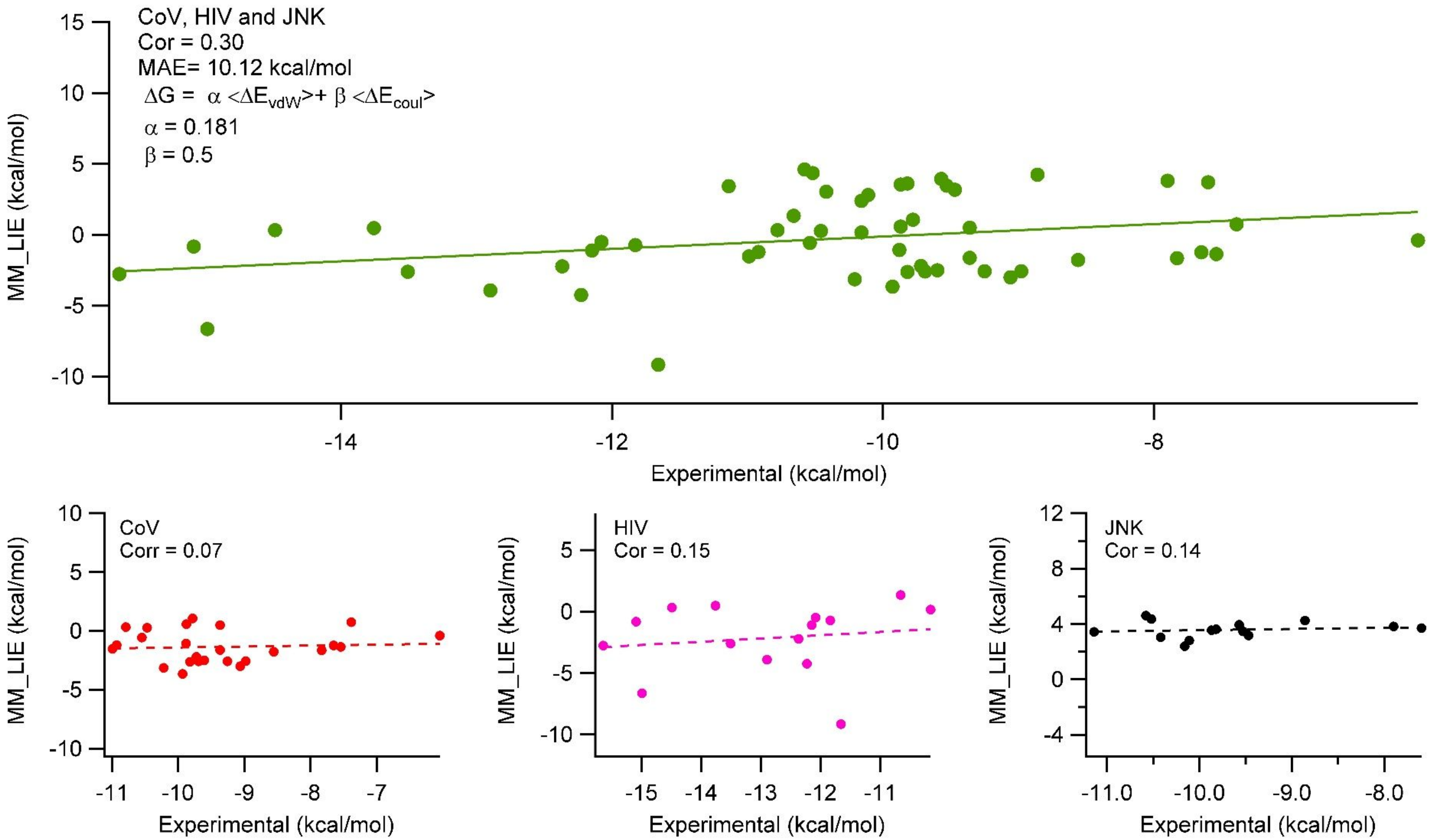

Figure S3: The LIE calculated by MM electrostatic and van der Waals interactions using equation (3). The preassigned parameters available in Gromacs software were used. The bottom plots show the correlation in each individual protein family.

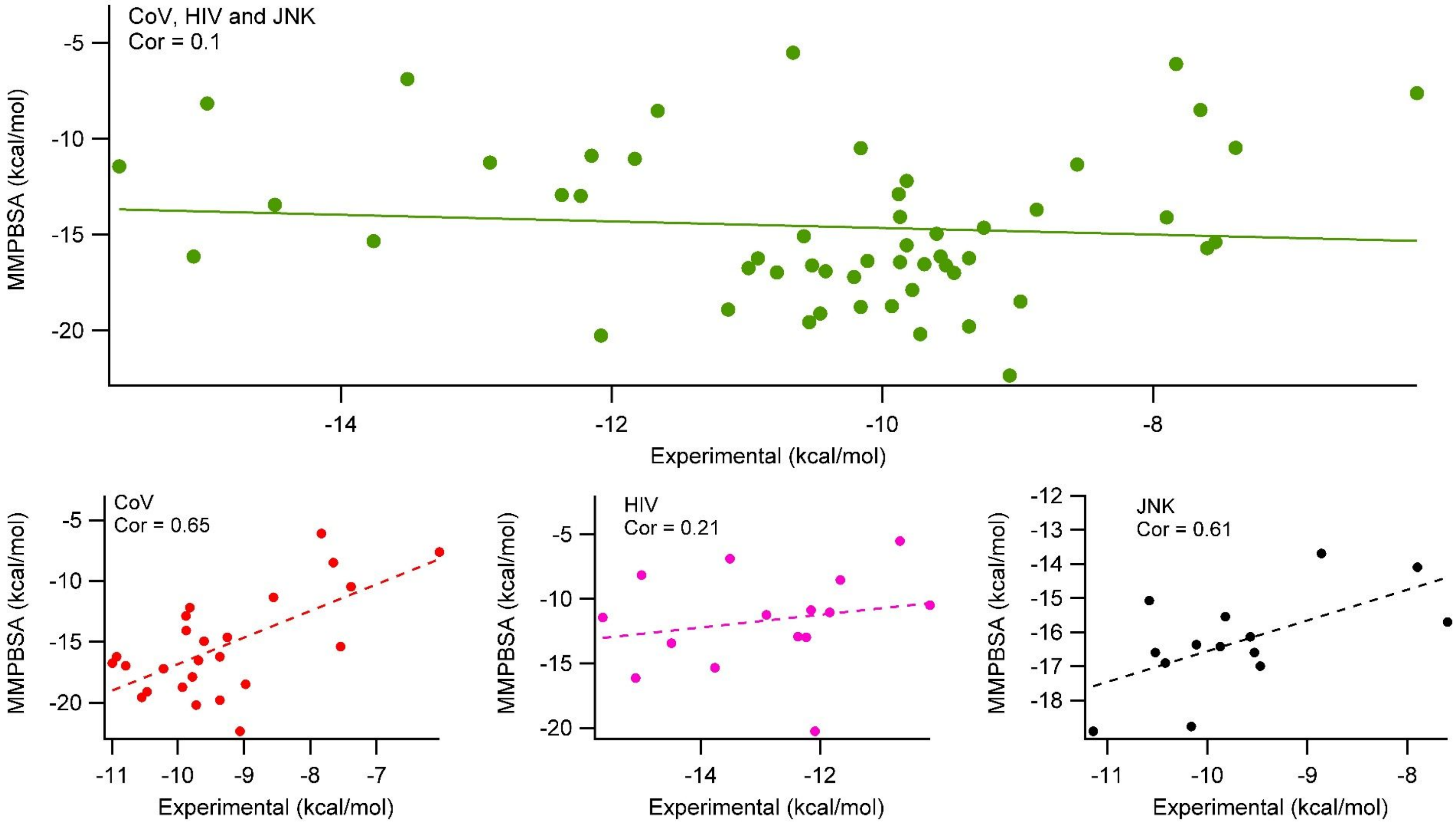

Figure S4: MMPBSA results show low correlation due to failure to predict BFEs in HIV-1 protease. The bottom plots show the correlation in each individual protein family.

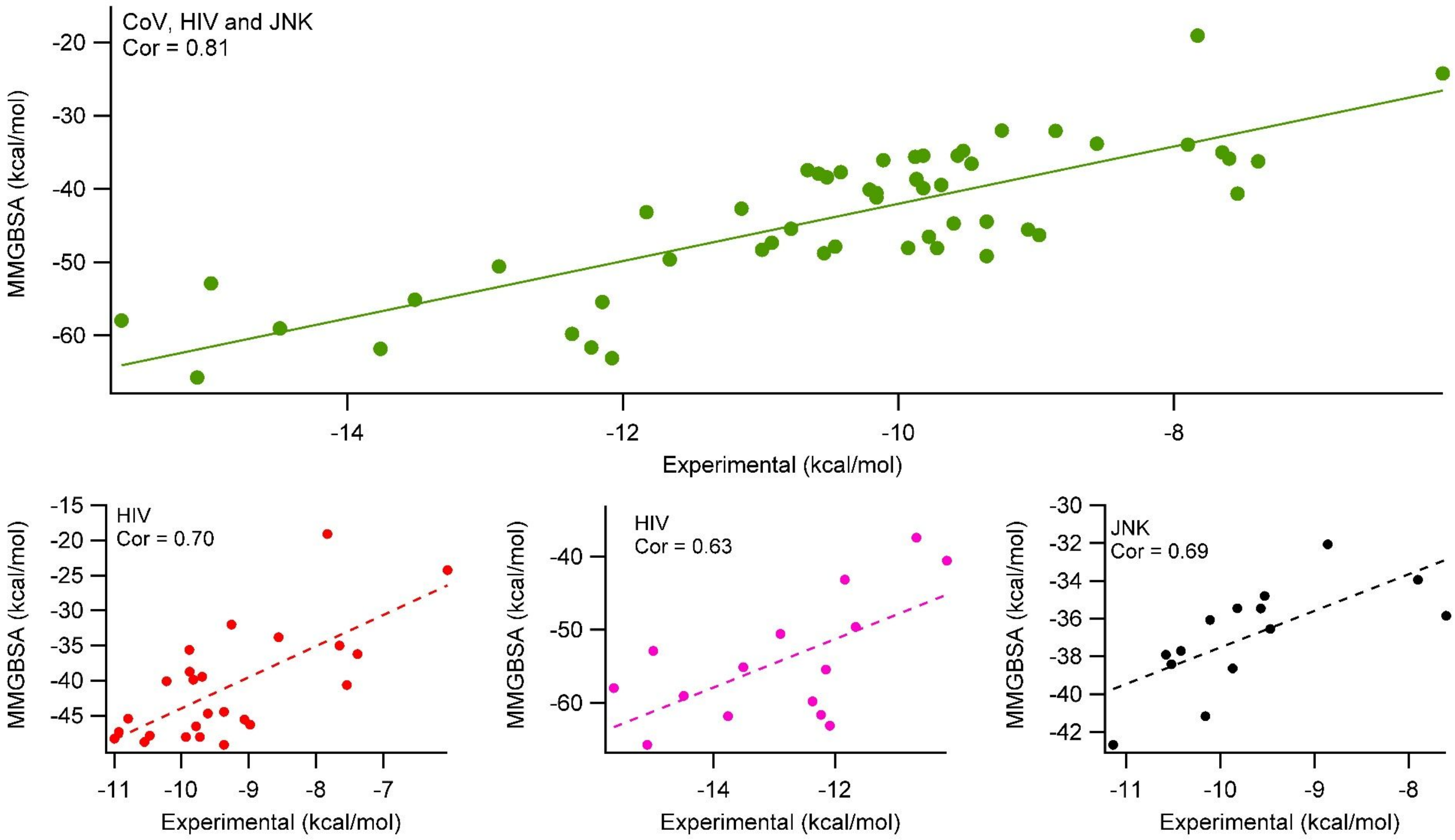

Figure S5: MMGBSA results showing higher correlation than LIE, but still lower than ANI_LIE. The bottom plots show the correlation in each individual protein family.

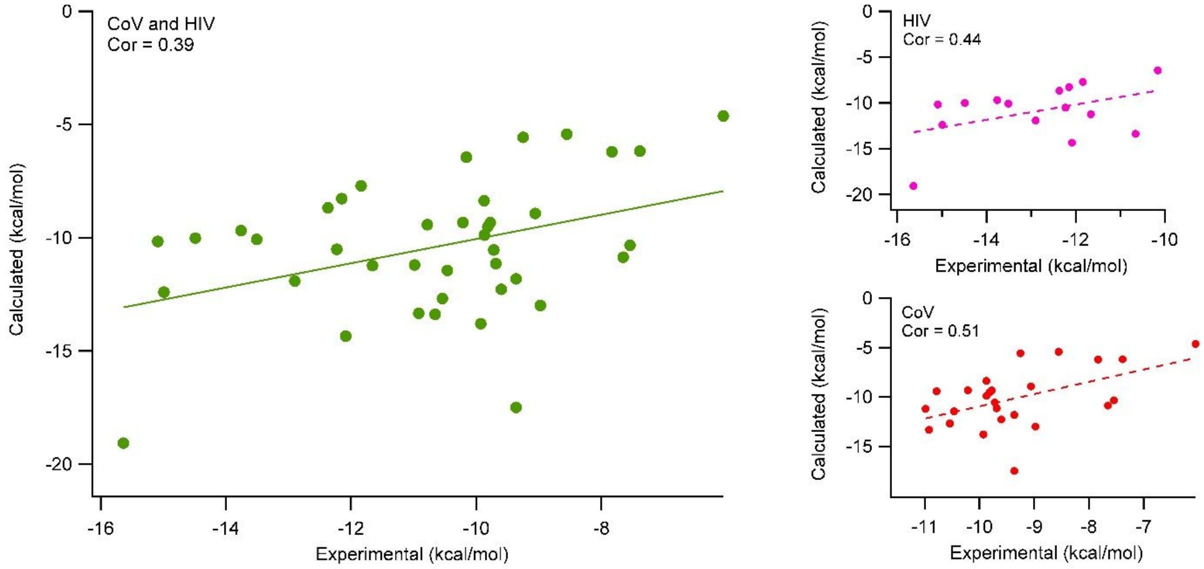

Figure S6: BAR results for CoV and HIV compared with experimental results when they are treated together or individually.

Table S2 Coefficient values derived from equations 4-7

Equation number	Method	α	β	γ (kcal/mol)
4	ANI_LIE	0	0.106± 0.016	-4.987 ± 0.881
5	ANID3_LIE	-0.025±0.028	0.124 ± 0.021	-5.569 ± 0.782
6	ANI_LIE	0	0.127±0.011	-5.111±0.480
7	ANID3_LIE	-0.035 ± 0.029	0.149 ± 0.021	-5.987 ± 0.806
7 (RS)	ANID3_LIE	-0.035±0.008	0.148±0.008	-5.978±0.230
α : van der Waals terms (LJ or D3); β:electrostatic term (ele or ANI); γ: intercept RS: Random sampling as validation (90% training bootstrapped by 100 different selections) and test (10%)				
